# A comparative study of the antioxidant and intestinal protective effects of extracts from different parts of Java tea (*Orthosiphon stamineus*)

**DOI:** 10.1002/fsn3.584

**Published:** 2018-02-06

**Authors:** Xuan Cai, Changfeng Xiao, Huiqin Xue, Huihui Xiong, Yiqiong Hang, Jianxiong Xu, Yonghong Lu

**Affiliations:** ^1^ Shanghai Shenfeng Animal Husbandry and Veterinary Science Technology Co., Ltd Shanghai China; ^2^ Institute of Animal Husbandry & Veterinary Science Shanghai Academy of Agricultural Sciences Shanghai China; ^3^ School of Agriculture and Biology Shanghai Jiao Tong University Shanghai China

**Keywords:** antioxidant, intestinal epithelial cell, Java tea, mice, *Orthosiphon stamineus*

## Abstract

The aim of this study was to compare the free radical scavenging ability and intestinal epithelial cell protective effects of Java tea (*Orthosiphon stamineus*) root extracts (ORE), stem extracts (OSE), and leaf extracts (OLE) to determine the potential of Java tea by‐products. The Java tea extracts were prepared using a standard water–ethanol method. The antioxidant activity and intestinal protective effects were tested by H_2_O_2_‐induced cell model and high‐fat diet‐induced mice model, respectively. The results showed that the total phenolic acid and flavonoid content and relative content were different in the ORE, OSE, and OLE. ORE had the highest total polyphenol and flavonoid content, the highest free radical scavenging rate, and the highest intracellular free radical scavenging rate. However, the yeast content in the ORE was lower than that in the OSE and OLE. All the Java tea extracts protected mouse intestine from high‐fat diet‐induced oxidative injury. This study indicates the potential of Java tea extracts as food or feed additives to protect the intestine from oxidative stress.

## 1 INTRODUCTION

The intestinal epithelium not only has the functions of nutrition digestion and absorption, but also is a barrier against antigens and pathogens (Suzuki, 2013). The intestine is exposed to a complex microenvironment that includes chyme, enterobacteria, various digestive juices, and immune factors. Imbalances in this microenvironment contribute to oxidative stress in the intestinal epithelium (Miranda‐Bautista, Bañares, & Vaquero, 2017).

Several plants and plant extracts, including green tea (Wan, Ling, Wang, & El‐Nezami, 2016), clove, and oak (Dudonné, Vitrac, Coutière, Woillez, & Mérillon, 2009), have strong antioxidant activity and intestinal protective effects. *Orthosiphon stamineus*, known as “Java tea,” is widely grown throughout South Asia, Australia, and southern China (Ameer, Salman, Asmawi, Ibraheem, & Yam, 2012). Furthermore, the tea is used in Southeast Asia and China for the treatment of kidney disease (Yam, Basir, Asmawi, & Ismail, 2007). Most importantly, several studies have confirmed that Java tea extracts exhibit strong antioxidant activity (Ameer et al., 2012). These findings suggest that Java tea may protect intestinal cells from oxidative stress.

However, the published reports evaluating *O. stamineus* extracts do not describe their effects in different organs. Only *O. stamineus* leaves and stems are routinely sold in Chinese markets, with the roots being discarded as a by‐product. This processing method is not only inconvenient for the consumer, but also a waste of *O. stamineus* resources. In fact, a previous study showed the stem and root of *O. stamineus* also possessed high antioxidant activities (Xue et al., 2016) and could be used as food or feed additives.

The aim of this study was to compare the main phenolic compounds with antioxidant activity from *O. stamineus* extracts, to determine the potential of these extracts as antioxidant additives and their protective effects on intestinal cells.

## 2 MATERIALS AND METHODS

### 2.1 Materials and plant extracts

*O. stamineus* was purchased from the Yulin Chinese herbal medicine market in Yulin, China. The plant was identified by Dr. H. B. Hu (Key Laboratory of Natural Drug Research and Development, Gannan Medical University), and a voucher specimen was retained in our laboratory for future reference. The roots, stems, and leaves were separated and then dried in a drying oven. The extracts were prepared using a water–ethanol method (Yam et al., 2007). Briefly, a 20 g dry powder of *O. stamineus* root and stem of leaves were subjected to an ultrasonic extractor at 50°C for 15 min in extracted with 1 L of 50% ethanol. The resulting *Orthosiphon stamineus* extracts were filtered and concentrated by applying vacuum rotary evaporation method. The concentrated liquid extract was freeze‐dried, and the powder stored at −20°C until use.

### 2.2 Animals and model treatment

Fifty male C57BL/6 mice weighing 18–20 g were purchased from Shanghai SLAC Laboratory Animal Co., Ltd. After being acclimated for 1 week, the mice were randomly divided into normal control (NC), high‐fat control (FC), root extract (R), stem extract (S), and leaf extract (L) groups, with 10 mice in each group. The mice were housed in standard cages under controlled temperature conditions (22 ± 2 °C) with a 12‐h light/dark cycle. The NC group received only a normal diet (D12450B, Research Diet Inc.) containing 4.3% fat, and other groups received a high‐fat diet (D12492; Research Diet Inc., New Brunswick, USA) containing 35% fat. The mice in the R, S, and T groups were orally administered *O. stamineus* root extracts (ORE), stem extracts (OSE), and leaf extracts (OLE) at a dose of 100 mg/kg body weight, while the mice in the NC and FC groups were orally administered saline. The oral administration lasted for 8 weeks. At the end of the study, blood samples were collected by eyeball removal. Jejunum is the longest segment in small intestine. In this study, jejunum samples of mice were washed immediately with ice‐cold PBS and stored at −80 °C prior to analysis. These experiments were carried out in accordance with local guidelines for the care of laboratory animals and were approved by the institution's ethics committee for research using laboratory animals.

### 2.3 Total phenolics and flavonoids analysis of *O. stamineus* extract

The total polyphenol content in the extracts was determined by the Folin–Ciocalteu method using gallic acid as the standard. Total flavonoids in the extract were determined using the method by Rana et al. (2015), with quercetin as the standard (Taga, Miller, & Pratt, 1984).

### 2.4 HPLC‐MS analysis

HPLC‐MS analyses were performed using an Acquity UPLC BEH‐C18 column (100 × 2.1 mm, 1.7 μm) at 45 °C with a mobile phase at a flow rate of 0.4 ml/min. The mobile phase consisted of 0.1% formic acid in water (phase A) and acetonitrile (phase B). The mobile phase was consecutively programmed as shown in Supporting Information. The mass spectra were obtained under both negative and positive ion modes, and the mass spectrometry conditions are shown in Supplementary Material. Data were processed by MultiQuant^™^ 2.1.1 Software (AB SCIEX, Framingham, USA).

### 2.5 Free radical scavenging activity in cell‐free systems

An oxygen radical absorbance capacity (ORAC) assay based on the scavenging of peroxyl radicals generated by 2,2′‐azobis(2‐methylpropionamidine) dihydrochloride (AAPH) was conducted. The assay was performed according to the method of Ou, Hampsch‐Woodill, and Prior (2001).

The DPPH radical scavenging activities of ORE, OSE, and OLE were determined according to Wu, Jiang, Jing, Zheng, and Yan (2017).

### 2.6 H_2_O_2_ challenge assay with IPEC‐J2 cell model

The H_2_O_2_‐induced IPEC‐J2 cell oxidative stress model was included according to a previous study (Cai et al., 2016). In this study, IPEC‐J2 cells were divided into five groups. The PBS group was the control group. In the test groups, 50 μg/ml of the ORE, OSE, or OLE was added to the final concentration for 24 hr before analysis, and then, 1 mmol/L H_2_O_2_ was added for 1 hr before testing. In the H_2_O_2_ group, 1 mmol/L H_2_O_2_ was added to the final concentration for 1 hr before the test. An intracellular total ROS assay and cell viability assay were performed.

The cell viability assay was performed using the cell counting kit method as described above. The inhibition ratio was calculated as:

Cell viability in relation to the control group = *A*_test_/*A*_control_ × 100%,

where *A*_test_ is the absorbance of the ORE, OSE, or OLE group, and *A*_control_ is the absorbance of the control group.

The intracellular free radical scavenging assay was performed using the 2′,7′ ‐dichlorofluorescein diacetate (DCFH‐DA) probe method.

### 2.7 Serum diamine oxidase (DAO) content

Serum was separated by centrifugation at 3,500 g for 15 min at 4°C. Serum concentrations of DAO were measured using a quantitative sandwich enzyme immunoassay technique according to the manufacturer's instructions (Cusabio Biotech Co., Wuhan, China).

### 2.8 Antioxidant analysis of jejunal homogenates

Jejunal homogenates (10% w/v) were prepared in cold PBS using homogenizer in ice and centrifugation at 4,000 g for 20 min at 4°C. The supernatants were diluted to the optimal content for detecting redox status. The protein content of homogenates was measured using the Coomassie Brilliant G‐250 method. The superoxide dismutase (SOD), glutathione peroxidase (GSH‐Px), and malondialdehyde (MDA) contents of jejunal homogenates were measured by colorimetry at absorbances of 550, 412, and 532 nm, respectively, according to the manufacturer's instructions (Nanjing Jiancheng Bioengineering Institute, Nanjing, China). All absorbances were measured by a microplate reader (Tecan Inc., Mannedorf, Switzerland).

### 2.9 Statistical analysis

All reaction mixtures were prepared in triplicate, and at least three independent assays were performed for each sample. All data are expressed as mean ± SEM. Data were subjected to one‐way ANOVA followed by Duncan's multiple range tests using SPSS version 17.0 software. A *p*‐value <.05 was considered to be statistically significant. Trends were reported when .05 < *p *< .1.

## 3 RESULTS

### 3.1 Yield

This study showed the yield of *O. stamineus* root extracts (ORE), *O. stamineus* stem extracts (OSE), and *O. stamineus* leaf extracts (OLE) to be 9.52%, 16.64%, and 16.70%, respectively.

### 3.2 Antioxidant content in *O. stamineus*

The amounts of total polyphenol in the ORE, OSE, and OLE were 266.25 ± 25.26, 82.92 ± 5.42, and 187.08 ± 28.42 μg gallic acid equivalent, respectively. The total flavonoids in the ORE, OSE, and OLE were 410.12 ± 25.84, 170.00 ± 29.52, and 367.44 ± 24.87 μg quercetin equivalent, respectively.

The major phenolic acids and flavonoids in the ORE, OSE, and OLE were detected by HPLC‐MS. The results (Table 1) show that there were significant differences in rosmarinic acid, caffeic acid, eupatorin, ursolic acid, and 3′‐hydroxy‐5,6,7,4′‐tetramethoxyflavone (3′‐OH‐TMF) content in the root, stem, and leaf extracts. The ursolic acid content was highest in the OSE; however, the sinensetin, eupatorin, 3′‐OH‐TMF, rosmarinic acid, and caffeic acid content were highest in the OLE.

**Table 1:** Content of the main antioxidant constituents of *Orthosiphon stamineus* root, stem, and leaf. Data expressed as means ± SEM (*n* = 3)

Compound	Root (mg/g)	Stem (mg/g)	Leaf (mg/g)
Sinensetin	0.097 ± 0.002	0.103 ± 0.001	2.719 ± 0.001
Eupatorin	0.184 ± 0.002	0.285 ± 0.003	4.731 ± 0.005
3′‐hydroxy‐5,6,7,4′‐tetramethoxyflavone	0.018 ± 0.000	0.025 ± 0.001	0.425 ± 0.013
Rosmarinic acid	18.426 ± 0.007	8.201 ± 0.051	19.861 ± 0.008
Caffeic acid	0.410 ± 0.005	0.259 ± 0.005	0.425 ± 0.010
Ursolic acid	17.642 ± 0.003	10.507 ± 0.001	0.422 ± 0.006

### 3.3 Antioxidant ability of *O. stamineus* in vitro

The ORAC and the 1,1‐diphenyl‐2‐picrylhydrazyl radical and 2,2‐diphenyl‐1‐(2,4,6‐trinitrophenyl) hydrazyl (DPPH) radical scavenging abilities of the *O. stamineus* extracts were analyzed to evaluate the antioxidant effects of the ORE, OSE, and OLE. The data revealed that ORE had the highest ORAC value. Additionally, 1 mg ORE was equivalent to 3.82 ± 0.16 mmol Trolox, and 1 mg OSE and OLE were equivalent to 1.80 ± 0.30 and 3.58 ± 0.16 mmol Trolox, respectively.

### 3.4 Antioxidant and cell protective effects of *O. stamineus* on IPEC‐J2 cells

As shown in Figure 2, the extracts of roots, stems, and leaves scavenged intracellular reactive oxygen species (ROS) and significantly increased cell viability under oxidative stress (*p *<* *.05). At a concentration of 50 μg/ml, the ORE had the highest intracellular ROS scavenging rate, but the OLE had the greatest cell viability increase (not significantly higher than that of the ORE, *p *>* *.05).

### 3.5 Serum DAO content

As shown in Figure 3, DAO concentrations were increased in high‐fat diet mice compared with the control mice (*p* < .05). *O. stamineus* root extract‐fed mice had DAO concentrations that were significantly lower than those of the high‐fat diet mice (*p* < .05) but still higher than those of control mice (*p* < .1). No significant differences in DAO concentration were found between ORE‐, OSE‐, and OLE‐treated mice (*p* > .05).

### 3.6 Antioxidant effect of *O. stamineus* on intestinal epithelia

Table 2 shows the MDA levels in jejunal homogenates from mice fed high‐fat diets significantly increased, while SOD and GSH‐Px activities decreased compared with those from control mice *(p* < .05). The homogenate MDA level was decreased by root, stem, and leaf extracts (*p* < .05), most significantly in the R group in comparison with the high‐fat group, but that of the S and L groups was still significantly higher than that of control group (*p* < .05). Table 2 also shows that extracts of *O. stamineus* did not alter the jejunal GSH‐Px activity (*p* > .1). The extracts of *O. stamineus* roots and leaves, but not stems, significantly increased mouse jejunal SOD activity (*p *< .05).

**Table 2:** Jejunal epithelium superoxide dismutase (SOD) and glutathione peroxidase (GSH‐Px) activities and malondialdehyde (MDA) content in mice of the normal control (NC), high‐fat control (FC), root extract (R), stem extract (S), and leaf extract (L) groups

Groups	SOD^a^ units/mg protein	GSH‐Px^b^ units/mg protein	MDA nmol/mg protein
NC	65.12 ± 2.11^a^	359.16 ± 25.21^a^	1.48 ± 0.18^c^
FC	56.55 ± 2.11^b^	312.67 ± 21.78^b^	2.61 ± 0.15^a^
R	63.47 ± 1.98^a^	343.56 ± 31.79^ab^	1.76 ± 0.12^bc^
S	60.57 ± 2.99^ab^	331.00 ± 19.78^ab^	2.12 ± 0.16^b^
L	62.33 ± 2.07^a^	341.07 ± 24.19^ab^	1.96 ± 0.10^b^

Mean values within a column with different superscript letters were significantly different (*p* < .05).

a One unit of SOD activity was defined as the amount required to inhibit the reduction in nitro blue tetrazolium by 50% of maximum inhibition in 1 mg tissue protein.

b One unit of GSH‐Px activity was defined as a decrease of μmol/L of GSH per 5 min for 1 mg protein at 37°C after subtraction of the nonenzymatic reaction.

## 4 DISCUSSION

Many reports have referred to the antioxidant activities and other pharmacologic effects of *O. stamineus* (Ameer et al., 2012). However, to our knowledge, the reports on *O. stamineus* to date have mainly related to leaf or stem extracts, with no literature on root extracts.

Phenolic acid and flavonoids were the main antioxidant compounds in the plant. More than 30 phenolic acids and flavonoids have been detected in *O. stamineus* (Ameer et al., 2012; Sumaryono, Proksch, Wray, Witte, & Hartmann, 1991). Our results show that both total polyphenol and total flavonoids were highest in the root extracts. However, the total polyphenol and total flavonoids yields from *O. stamineus* leaves are higher than those from the roots.

The present study shows rosmarinic acid to be the most abundant phenolic acid of *O. stamineus* in both leaves and roots. This finding is consistent with the reports by Akowuah, Zhari, Norhayati, and Sadikun (2004) and Lee, Peng, Chang, Huang, and Chyau (2013). The study by Lee et al. (2013) showed rosmarinic acid to be the major contributor to the antioxidant activities of *O. stamineus*. Interestingly, the present study showed that ursolic acid was also present at very high levels in *O. stamineus* and that the ursolic acid content of roots and stems was much higher than that of leaves (Table 1). Ursolic acid is a well‐known anticancer agent (Chen et al., 2015), while rosmarinic acid shows cellular protective effects (Nabavi et al., 2015). These results suggest that ORE, OSE, and OLE may display different bioactivities on cellular proliferation (Figure 2); however, the mechanism by which *O. stamineus* extracts regulate cellular processes needs further study.

**Figure 1:**
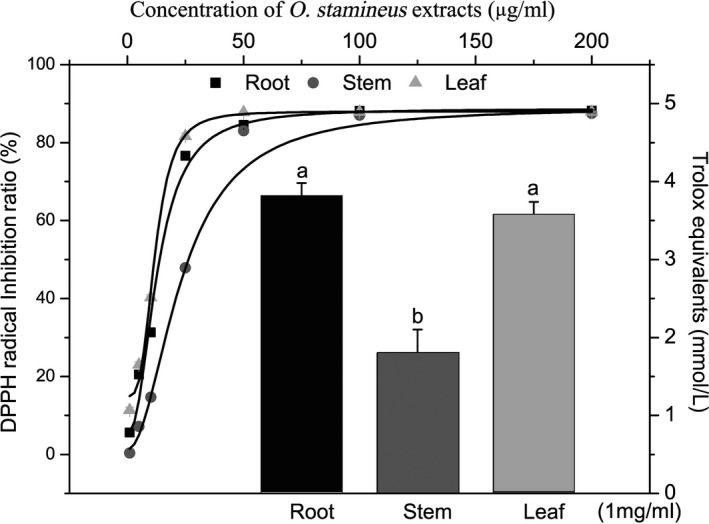
Antioxidant effect of *Orthosiphon stamineus* extracts. The left Y and top X showed the DPPH radical inhibition ratio by the ORE, OSE, and OLE in different concentrations

This study showed the antioxidant effects of *O. stamineus* extract vary significantly between different organs. The ORAC values of the root and leaf extracts were higher than those in the study by Yam et al. (2007) (2.73 mmol/L), but that of the stem extract was lower than that obtained by Yam et al. It may be that “*O. stamineus* leaves” in markets usually contain stems, which results in a lower ORAC value than that found in the pure leaf extract. The *O. stamineus* extracts showed concentration‐dependent DPPH radical scavenging activity. The root extract and leaf extract had very similar DPPH radical scavenging curves, while the stem extract showed the lowest DPPH radical inhibition activity. The IC50 values of the ORE, OSE, and OLE were 13.72, 26.55, and 11.34 μg/ml, respectively (Figure 1). The cell model studies also yielded similar results: Root extracts showed the highest intracellular ROS scavenging rate, whereas stem extracts showed the lowest intracellular ROS scavenging rate. The in vivo study confirmed these results, with the R group of mice having the lowest jejunal MDA content, and the S group the highest. MDA is one of the key toxic products of lipid peroxidation, a process that disrupts membrane structure and slows cellular metabolism (Moon & Shibamoto, 2009). The data in Table 2 show the high‐fat diet induced a high‐lipid peroxidation rate in mice and that *O. stamineus* extracts reduced this effect. However, the mechanism by which this occurs does not appear to relate to the levels of antioxidative enzymes such as SOD or GSH‐Px (Table 2). The findings of a study by Choi et al. (2013) may partly account for this: *O. stamineus* extracts increased leptin expression in mice, and leptin decreased tissue MDA levels (Hacioglu, Algin, Pasaoglu, Pasaoglu, & Kanbak, 2005). This is an interesting topic, and more data are still needed to confirm this hypothesis. Our results show that *O. stamineus* extracts can protect intestine from oxidative stress and that not only the leaf but also the stem and root have good oxygen radical and nitrogen radical scavenging activity.

**Figure 2:**
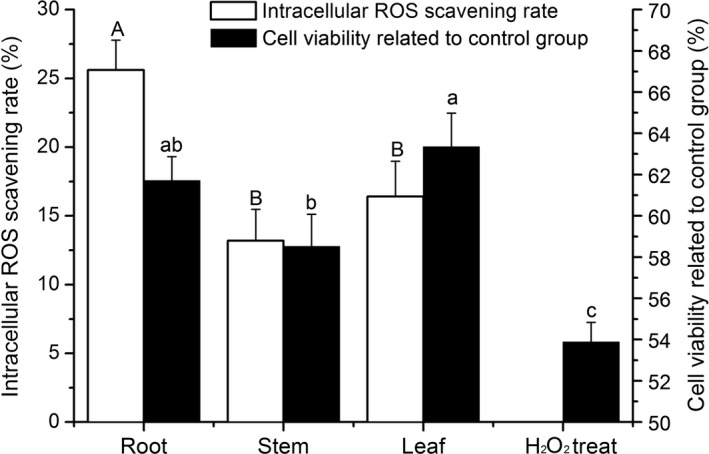
Intracellular ROS scavenging and cell viability were increased by the *Orthosiphon stamineus* extracts. Different letters represent significant differences (*p* < .05)

**Figure 3:**
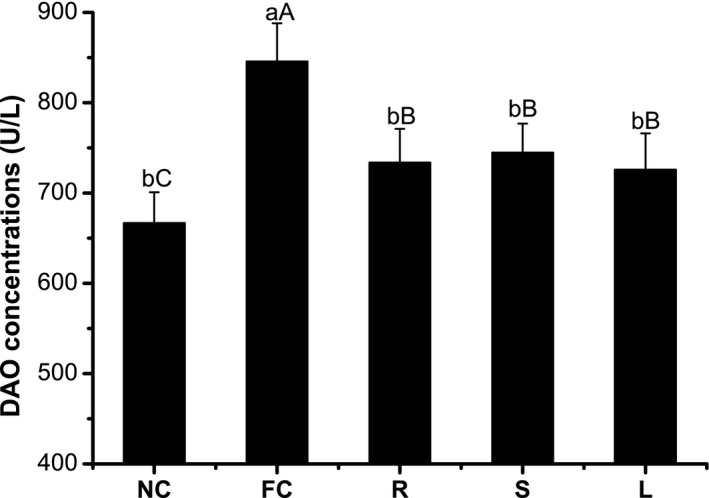
Effect of *Orthosiphon stamineus* extracts on mice serum DAO concentrations. All mice were feeded for 8 weeks by normal diet (control group) or high‐fat diet (other groups), ORE, OSE, and OLE group mice i.g. administrated with ORE, OSE, and OLE at a dose of 100 mg/kg body weight. Different small letters represent significant differences (*p* < .05), and different capitals represent there were trends of differences (*p* < .1)

IPEC‐J2 is a nontumorigenic epithelial cell line and is a suitable oxidative stress model (Cai et al., 2016). Figure 2 shows that extracts of *O. stamineus* significantly promoted IPEC‐J2 cell viability under conditions of H_2_O_2_‐induced oxidative stress. This implies that *O. stamineus* extracts could protect intestine from stress. The mouse experiment confirms this theory. Figure 3 shows that orally administered *O. stamineus* significantly reduced DAO concentrations compared with the FC group. DAO is an enzyme found in high concentrations in the intestinal mucosa but in low concentrations in plasma. Plasma DAO concentrations significantly increase following intestinal mucosal damage. Thus, plasma DAO levels can serve as a marker of mucosal integrity (Çakmaz et al., 2013). This means that orally administered *O. stamineus* could protect intestinal mucosa from stress‐induced damage, with no significant difference between the effects of the root, stem, and leaf extracts.

This is the first report to compare *O. stamineus* leaf, stem, and root standard water–ethanol extracts. Our results show that the root, stem, and leaf of *O. stamineus* contained similar phenolic acid and flavonoid compounds, but that the total and relative phenolic acid and flavonoids content of each were different. *O. stamineus* extracts scavenged intracellular free radicals and protected IPEC‐J2 intestinal epithelial cells from H_2_O_2_‐induced oxidative stress injury. The ORE had the highest polyphenol and flavonoids content, ORAC value, and DPPH radical scavenging rate. The ORE also showed the highest intracellular free radical scavenging rate, but the yeast content in the ORE was lower than that in the OSE and OLE. Therefore, the establishment of a highly effective extraction method for *O. stamineus* roots is necessary. These results indicate that Java tea by‐products have potential as a food or feed additive for protecting the intestine from oxidative stress. If we could separate the leaves and stems of *O. stamineus* and process the leaves to drink while processing the stems and roots as food or feed additives, we could not only offer a better drink for human consumption, but also produce a large amount of raw material for animal feed or natural food additives.

## CONFLICT OF INTERESTS

None declared.
