# Supplementary material for: A comparative study of the antioxidant and intestinal protective effects of extracts from different parts of Java tea (Orthosiphon stamineus)
Source: Food Sci Nutr. 2018 Feb 6;6(3):579–84. doi: 10.1002/fsn3.584 (PMC5980324; doi:10.1002/fsn3.584)
Supplement: Supplementary file 1 [file FSN3-6-579-s001.docx]

S2 Mice body weight change and leptin concentration in blood

The mice body weight in this study were shown in Fig. S2-1. All the groups feeded with OS extracts (L, S, R group) showed no significantly difference with FC group, but the body weight of all the groups feed with high-fat diet (FC, L, S, R group) significantly higher than the group feed with normol diet (NC group).


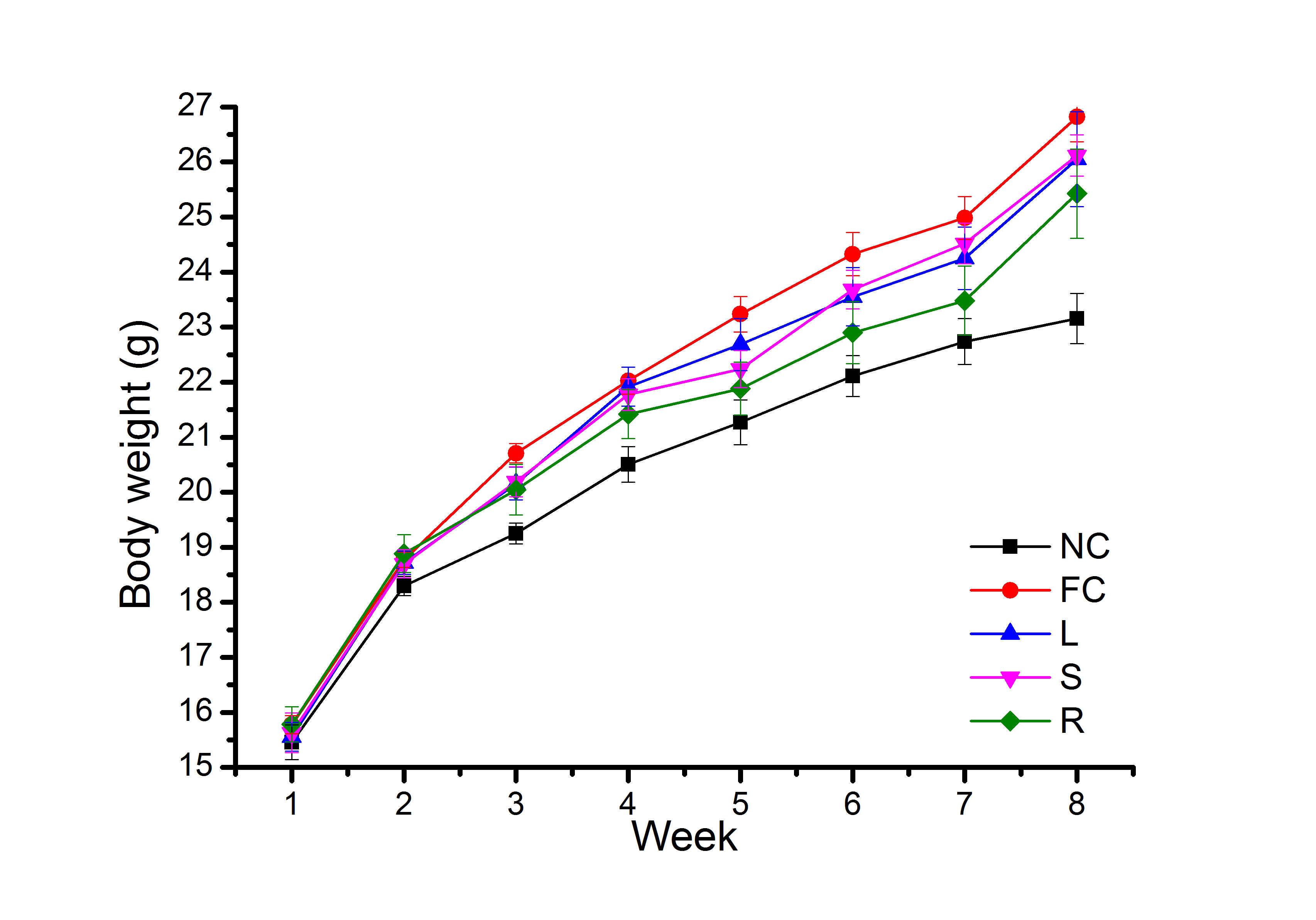


**Fig. S2-1 Mice body weight change**


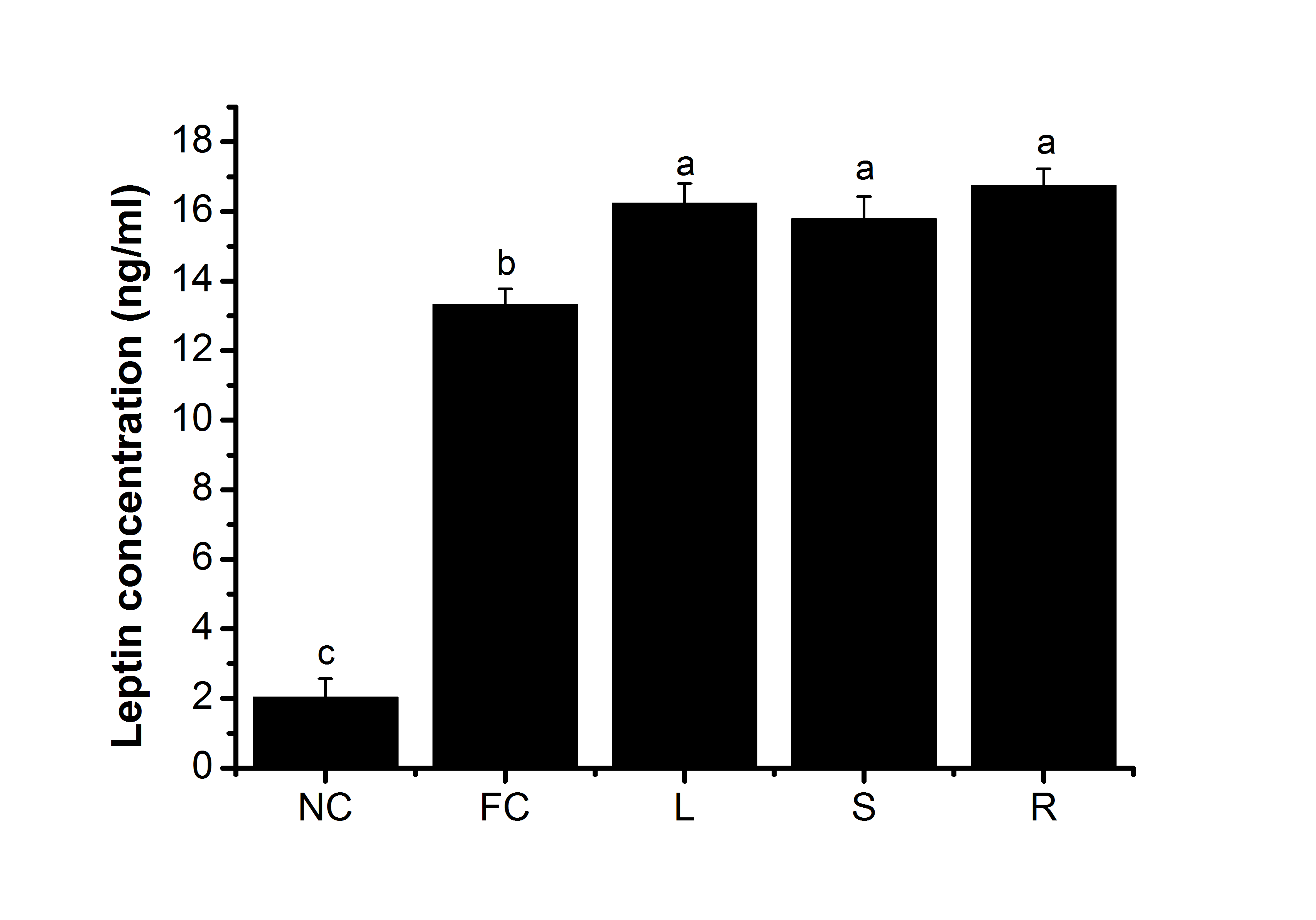


**Fig. S2-2 Serum leptin concentration in mice.** Values that do not share a common superscript are significantly different at p < 0.05.

The leptin concentration in blood were analyzed by ELISA method in this study. All operations are performed according to the operation manual (Boster Biological Technology co.Itd, Wuhan, China). Data (Fig. S2-2) showed high-fat diet fed significantly increase serum leptin concentration (p <0.05) and OS extracts intragastric administration mice (L, S and R Group) showed higher (p <0.05) leptin concentration than that saline intragastric administration mice (FC Group). The serum leptin concentration between L, S and R Group showed no difference in this study (p > 0.05). This result indicated OS extracts may not alter the animal body weight but stimulate leptin expression. These were consistent with the study by Choi et al.([Choi et al., 2013](#_ENREF_1)).

**Refrence**

Choi, Y. J., Park, S. Y., Kim, J. Y., Won, K. C., Kim, B. R., Son, J. K., . . . Kim, Y. W. (2013). Combined Treatment of Betulinic Acid, a PTP1B Inhibitor, with Orthosiphon stamineus Extract Decreases Body Weight in High-Fat-Fed Mice. *Journal of Medicinal Food, 16*(1), 2-8. doi: 10.1089/jmf.2012.2384
