# Supplementary material for: A comparative study of the antioxidant and intestinal protective effects of extracts from different parts of Java tea (Orthosiphon stamineus)
Source: Food Sci Nutr. 2018 Feb 6;6(3):579–84. doi: 10.1002/fsn3.584 (PMC5980324; doi:10.1002/fsn3.584)
Supplement: Supplementary file 2 [file FSN3-6-579-s002.docx]

Supplementary material

## HPLC-MS analysis

All analyses were performed using a Acquity UPLC BEH-C18 column (100×2.1 mm, 1.7μm) at 45℃with a mobile phase at a flow rate of 0.4 mL/min. The mobile phase was 0.1% fomic acid in water (phase A) and acetonitrile (phase B). The mobile phase was consecutively programmed as Table1:

Table 1 The mobile phase program

| Time(min) | Flow | Rate | %A | %B |
| --- | --- | --- | --- | --- |
| 1 | Initial | 0.4 | 99 | 1 |
| 2 | 1 | 0.4 | 99 | 1 |
| 3 | 5 | 0.4 | 5 | 95 |
| 4 | 7 | 0.4 | 5 | 95 |
| 5 | 7.1 | 0.4 | 99 | 1 |
| 6 | 9 | 0.4 | 99 | 1 |

The mass spectra were collected under both negative and positive ion modes and the mass spectrometer conditions were shown at Table 2. Data processed by MultiQuant™ 2.1.1 Software.

Table 2 Mass Spectrometer Conditions

| Parameter | Value | |
| --- | --- | --- |
|  | Positive | Negative |
| CUR | 45 | 45 |
| GS1 | 45 | 45 |
| GS2 | 45 | 45 |
| IS | 5000 | 4500 |
| TEM | 550 | 550 |
| CE | 50 | -35 |
| CXP | 12 | -12 |
| DP | 100 | -100 |
| EP | 10 | -10 |
